# Improvement of Resveratrol Permeation through Sublingual Mucosa: Chemical Permeation Enhancers versus Spray Drying Technique to Obtain Fast-Disintegrating Sublingual Mini-Tablets

**DOI:** 10.3390/pharmaceutics13091370

**Published:** 2021-08-31

**Authors:** Giulia Di Prima, Giuseppe Angellotti, Amalia Giulia Scarpaci, Denise Murgia, Fabio D’agostino, Giuseppina Campisi, Viviana De Caro

**Affiliations:** 1 Dipartimento di Scienze e Tecnologie Biologiche Chimiche e Farmaceutiche (STEBICEF), University of Palermo, Via Archirafi 32, 90123 Palermo, Italy; giuseppe.angelloti@unipa.it (G.A.); amaliagiulia.scarpaci@unipa.it (A.G.S.); denise.murgia@unipa.it (D.M.); viviana.decaro@unipa.it (V.D.C.); 2 Dipartimento di Discipline Chirurgiche, Oncologiche e Stomatologiche, Università degli Studi di Palermo, 90127 Palermo, Italy; giuseppina.campisi@unipa.it; 3 Istituto per lo Studio degli Impatti Antropici e Sostenibilità dell’Ambiente Marino, Consiglio Nazionale delle Ricerche (IAS—CNR), Campobello di Mazara, 91021 Trapani, Italy; fabio.dagostino@cnr.it

**Keywords:** resveratrol, sublingual mucosa, spray drying, chemical permeation enhancers, menthol, lysine, urea, Transcutol^®^, sodium dodecyl sulfate, sodium dehydrocolate

## Abstract

Resveratrol (RSV) is a natural polyphenol with several interesting broad-spectrum pharmacological properties. However, it is characterized by poor oral bioavailability, extensive first-pass effect metabolism and low stability. Indeed, RSV could benefit from the advantage of the sublingual route of administration. In this view, RSV attitudes to crossing the porcine sublingual mucosa were evaluated and promoted both by six different chemical permeation enhancers (CPEs) as well as by preparing four innovative fast-disintegrating sublingual mini-tablets by spray drying followed by direct compression. Since RSV by itself exhibits a low permeation aptitude, this could be significantly enhanced by the use of CPEs as well as by embedding RSV in a spray-dried powder to be compressed in order to prepare fast-disintegrating mini-tablets. The most promising observed CPEs (menthol, lysine and urea) were then inserted into the most promising spray-dried excipients’ compositions (RSV-B and RSV-C), thus preparing CPE-loaded mini-tablets. However, this procedure leads to unsatisfactory results which preclude the possibility of merging the two proposed approaches. Finally, the best spray-dried composition (RSV-B) was further evaluated by SEM, FTIR, XRD and disintegration as well as dissolution behavior to prove its effectiveness as a sublingual fast-disintegrating formulation.

## 1. Introduction

Nowadays, the use of natural compounds for prophylactic, co-adjuvant and therapeutic purposes receives a broad consensus. Indeed, nutraceuticals are generally characterized by less side effects and lower production costs than synthetic drugs. In addition, the actual and always-growing knowledge in the fields of pharmacology, physiology and immunonutrition highlights the effectiveness of natural compounds as alternative medicines or as helpful adjuvant therapeutic agents combined with conventional treatments. This is particularly true for resveratrol (RSV), a nutraceutical that exhibits interesting and broad-spectrum pharmacological activities that make it a promising candidate for potential applications in different fields [1,2,3]. RSV (active form: 3,4′,5-trihydroxy-trans-stilbene) is a phenolic phytoalexin obtained from red grapes, red wine and wild berries, as well as by both chemical and biotechnological synthesis [4]. Thanks to its molecular structure, RSV is able to bind different biomolecules, thus causing a wide range of potential therapeutic effects [5]. In particular, RSV has shown anti-inflammatory, antioxidant, anticancer, cardioprotective and immunomodulatory properties; it seems to also be effective in treating neurodegenerative disorders and diabetes [1,6,7]. Furthermore, recent studies have also shown a promising antiviral activity that could result in being useful in the treatment of respiratory hyperinflammatory infections, such as COVID-19 [8,9,10]. Despite its great potentiality, RSV is characterized by unfavorable physicochemical properties (e.g., aqueous solubility ≈ 0.05 mg/mL), instability (due to light exposure, alkaline pH and high temperature) and poor oral bioavailability (< 1% due to extensive first-pass effect metabolism) [11,12,13,14,15]. Therefore, it is essential to find novel, alternative and innovative administration strategies in order to benefit from RSV’s therapeutic potential. In particular, the sublingual mucosa could be a useful alternative administration route as it allows direct absorption into the systemic circulation and bypasses the first-pass hepatic metabolism, thus increasing the overall bioavailability. The sublingual mucosa is a thin, non-keratinized tissue characterized by a high surface area (26.5 ± 4.2 cm^2^ if considering the sum of the ventral surface of the tongue together with the mouth floor) and extensive blood supply, which could assure fast onset of action and high plasma concentration [16,17,18]. When considering the transmucosal pathway, it might be useful to evaluate all the available strategies with which to enhance drug permeation across the tissues. These strategies could include conventional chemical enhancement and innovative technological approaches. The most frequently used conventional strategy involves the use of chemical permeation enhancers (CPEs), which are molecules that are able to effectively interact with the epithelia and transiently as well as reversibly modify their structure while promoting drug absorption [19,20,21]. The CPEs could be classified as follow: surfactants (e.g., sodium lauryl sulfate or sodium dodecyl sulfate), bile acids and their salts (e.g., sodium dehydrocolate), co-solvents (e.g., polyethylene glycol), positively charged amino acids (e.g., lysine and arginine), terpenes (e.g., menthol and limonene) and other molecules (e.g., urea and diethylene glycol monoethyl ether, also known as Transcutol^®^) [22,23,24,25,26,27,28]. On the other hand, the accurate design of innovative drug delivery systems (DDSs) could represent an alternative approach to promote drug permeation. In particular, fast-disintegrating sublingual tablets are able to quickly disintegrate, once in contact with the saliva, before being swallowed. This leads to high drug concentration in situ and rapid absorption through the highly permeable sublingual mucosa. Moreover, fast-disintegrating sublingual tablets should be extremely patient-friendly [16,29]. There are several technologies for the manufacturing of sublingual tablets, including spray drying, freeze drying, sublimation, direct compression and granulation processes. Among these, the spray drying technique represents an innovative strategy to obtain optimal pharmaceutical powders, characterized by homogeneous size distribution, shape, porosity, density, drug loading and chemical composition, which can be directly compressed in order to easily prepare sublingual tablets [30,31].

Based on these considerations, this study aims to evaluate the effectiveness of both the CPEs used and the innovative spray drying approach to enhance RSV sublingual permeation, and subsequently merge the two mentioned strategies in order to propose an optimized fast-disintegrating sublingual formulation. Firstly, the ability of RSV to cross the sublingual mucosa in the presence of six different CPEs was evaluated in order to identify the best ones. Contextually, by the spray drying technique, RSV-loaded pharmaceutical powders were developed to finally produce RSV-loaded mini-tablets. Spray-dried powders also containing CPEs were then obtained and evaluated in the form of mini-tablets as well. Finally, the best composition in terms of increased solubility and sublingual permeation enhancement of RSV was further characterized to prove its effectiveness as a sublingual fast-disintegrating formulation.

## 2. Materials and Methods

### 2.1. Materials

Trans-resveratrol (RSV) was purchased from A.C.E.F. Spa (Fiorenzuola D’Arda, Italy). Polyvinylpyrrolidone K90 (PVP K90), propylene glycol, sorbitol and lysine hydrochloride were obtained from Farmalabor (Canosa di Puglia, Italy). Urea, sodium dodecyl sulfate and menthol were supplied by Carlo Erba (Milan, Italy). Transcutol^®^ was purchased from Sigma-Aldrich Chemie (Steinheim, Germany). β-cyclodextrin (β-CD) was obtained from Roquette Italia S.P.A (Cassano Spinola, AL, Italy). Trehalose was purchased from Hayashibara Shoij (Okayama, Japan). Polyethylene glycol 200 (PEG_200_) was purchased from Fluka (Rodano, Italy) and trifluoroacetic acid (TFA) was obtained from Merk (Darmstadt, Germany). A citrate buffer solution of 10 mM (pH 5.5) was prepared by dissolving 2.052 g of anhydrous sodium citrate and 0.636 g of citric acid monohydrate in 1 L of distilled water. Isotonic saline solution (0.9 % *w*/*v*) was prepared by dissolving 9 g of sodium chloride (NaCl) in 1 L of distilled water. The isotonic saline solution containing 5% *w*/*v* of trehalose was prepared by dissolving 9 g of NaCl and 50 g of trehalose in 1 L of distilled water. All chemicals and solvents were purchased from VWR International (Leuven, Belgium), were of analytical grade and were used without further purification. Porcine sublingual mucosae were kindly supplied by the Municipal Slaughterhouse of Villabate (Palermo, Italy).

### 2.2. Methods

#### 2.2.1. CPEs to Promote RSV Sublingual Permeation

##### Preliminary Stability Assay of RSV

Considering the previously reported RSV solubility experiments and the literature data, stability studies were performed to assess the optimal experimental conditions for further analysis [4,13]. RSV (0.1% *w*/*v*) was dissolved in a citrate buffer solution (pH 5.5) and PEG_200_ mixture (80:20 *v*/*v*), with or without N-acetylcysteine (NAC) 0.2% (*w*/*v*). Each solution (10 mL) was kept in the dark at 37 ± 0.5°C under continuous stirring (Heidolph MR3001K Hotplate Stirrer with Heidolph EXT3001 Temperature Probe, Heidolph Instruments, Schwabach, Germany) for 6 h. Every 30 min samples (500 μl) were withdrawn and analyzed to determine RSV concentration by both HPLC and UV-Vis methods. Each experiment was performed in triplicate. For the UV-Vis analysis a Shimadzu 1700 instrument (Japan) was used, and RSV was evaluated at λ = 305 nm. RSV standard solutions in the citrate buffer solution (pH 5.5) and PEG_200_ mixture (80:20 *v*/*v*) were prepared to construct the calibration curve (concentration range: 0.00002–0.0080 mg/mL; y = 112.56x − 0.001; and R = 0.999). For HPLC-DAD analysis, a HPLC Agilent 1260 Infinity Instrument equipped with a Quaternary Pump G1311B, a Diode Array Detector 1260 Infinity II and a computer integrating apparatus (OpenLAB CDS ChemStation Workstation) was used (injected volume: 20 μl; column temperature: 25 ± 1°C). Chromatographic separation was achieved on a reversed-phase column, Ace^®^ Excel Super C18 (5U, 100A, size 125 × 4.60 mm), and employed a 0.1% (*v*/*v*) TFA solution in water (solvent A) and acetonitrile (solvent B) by using the following time program: 0–2 min A:B = 70:30; 2–8 min A:B = 20:80; and 8–12 min A:B = 70:30. The flow rate was set at 1 mL/min and the UV wavelength at 305 nm. In these conditions, the retention time of RSV was 5.3 min. Standard curves were used for the quantification of integrated areas under the peaks. The calibration curves were performed in the concentration range of 0.0001–0.01 mg/mL by injecting RSV standard solutions into methanol. Samples were injected after proper dilution. LOD and LOQ were 0.017 and 0.050 μg/mL, respectively. HPLC data were highly reproducible and linearly related to concentration (y = 87,308.09x; R = 0.999).

##### Ex Vivo Evaluations

- Tissue Preparation

Porcine tongues were collected from freshly slaughtered domestic 12-month-old pigs (intended for human consumption) and immediately transferred to the laboratory in a refrigerated transport box within 1 h from animal sacrifice. The animal tissues were then washed in PBS, pH 7.4, and excised in order to remove any excess of tissue. The samples were placed in trehalose solution (5% *w*/*v* in isotonic buffer), left for 1 h and then kept at −80°C for at least one week. Before the ex vivo permeation studies, specimens were washed for 1 h in isotonic solution and then subjected to thermal shock in order to obtain the sublingual mucosa. Briefly, tissue samples were dipped for approximately 2 min in a prewarmed isotonic solution (70°C), and the mucosa (80 ± 8 μm) was then carefully peeled off from the adipose tissue and submucosal connective tissue manually [32,33]. Mucosal specimen thickness was measured by using a digital micrometer (VWR International, Milano, Italy).

- Ex Vivo Permeation Assay

To evaluate RSV permeation throughout porcine sublingual mucosa, vertical Franz-type diffusion cells (PermeGear, flat flange joint, 9 mm orifice diameter and 15 mL acceptor volume, SES GmbH–Analysesysteme, Bechenheim, Germany) were used as a two-compartment open model. The obtained sublingual mucosa was equilibrated in the isotonic solution overnight at room temperature to remove all the biological matter which could interfere with the drug analysis. Afterwards, appropriate sections of mucosa were mounted between the acceptor and the donor chambers of Franz cells respectively filled with citrate buffer solution (pH 5.5) and 3% β-CD (*w*/*v*) citrate buffer (pH 5.5) and left to equilibrate at 37 ± 0.5 °C for 15 min. Hereafter, the citrate buffer was removed from the donor compartment and replaced with 1 mL of the solutions to be tested. At scheduled time intervals (15 min), samples (0.5 mL) were withdrawn from the acceptor compartment and immediately replaced with fresh acceptor fluid to maintain the sink conditions. Each experiment was carried out at 37.0 ± 0.5°C, under continuous stirring, in the dark conditions for 3 h and repeated 6 times. The donor solutions analyzed were the following: RSV solution 1 mg/mL in a citrate buffer solution (pH 5.5) and PEG_200_ mixture (80:20 *v*/*v*); RSV 1 mg/mL and chemical permeation enhancer (CPE) 0.2 mg/mL (RSV:CPE = 5:1) solution in a citrate buffer solution (pH 5.5) and PEG_200_ mixture (80:20 *v*/*v*). The employed CPEs were sodium dodecyl sulfate (SDS), lysine hydrochloride (LYS), menthol (M), urea (U), sodium dehydrocolate (SDC) and Transcutol^®^ (T). The amount of RSV permeated was quantified by UV-Vis analyses, after proper dilution of the recovered samples, by using a Shimadzu 1700 instrument (Japan). RSV standard solutions in 3% β-CD (*w*/*v*) citrate buffer (pH 5.5) were prepared to construct the calibration curve (λ = 305 nm; concentration range: 0.0005–0.0075 mg/mL; y = 123.72x − 0.02; and R = 0.999). Results are reported as means ± standard error (SE).

- Evaluation of RSV Amount Entrapped in the Sublingual Tissue

At the end of each permeation experiment, the Franz cells were disassembled and the porcine mucosa was collected to evaluate the amount of RSV entrapped in the tissue. Each sublingual mucosa specimen was washed with distilled water (3 × 2 mL) to remove any residue on its surface, and the RSV was then extracted by treating the tissue for 2 min with warmed (50–60°C) methanol (2 mL). The extraction was repeated 2 times. The extraction liquors were collected, transferred to a 10 mL flask and brought to volume with methanol. The amount of drug extracted was quantified by UV-Vis analysis, after proper dilution of the recovered samples, by using a Shimadzu 1700 instrument (Japan). RSV standard solutions in methanol were prepared to construct the calibration curve (λ = 305 nm; concentration range: 0.001–0.005 mg/mL; y = 143.38x + 0.04; and R = 0.999). Results are reported as means ± standard error (SE).

##### Determination of the Biopharmaceutical Parameters: J_s_, Kp, t_lag_, De and Ac

Drug flux (J_s_) through the sublingual tissue was calculated at the steady state per unit area by linear regression analysis of permeation data following the relationship:

(1) $$J_{s}=\frac{Q}{A\times \;t}\;(\mathrm{mg}/{\mathrm{cm}}^{2}\;\times \;h^{-1}\;\mathrm{or}\;\mu g/{\mathrm{cm}}^{2}\;\times \;h^{-1})$$

where Q is the amount of RSV (mg or μg) permeated during the time interval, t (h), and A is the area of sublingual mucosa in contact with RSV solutions (0.636 cm^2^). At the steady state J_s_ is equal to the slope of the straight line obtained. The constant of permeability (K_p_) was then calculated by the relationship:

(2) $$K_{p}=\frac{J_{s}}{C_{d}}(\mathrm{cm}/h)$$

where C_d_ is the starting RSV concentration loaded into the donor compartment. The t_lag_ (min) was determined from the interception of the tangent to the linear portion of the permeation profile with the *x*-axis.

Similarly, the amount of drug entrapped (De) per unit area in the mucosal tissue was calculated at the end of each experiment following the relationship:

(3) $$D_{e}=\frac{Q_{T}}{A}\;(\mathrm{mg}/{\mathrm{cm}}^{2}\;\mathrm{or}\;\mu g/{\mathrm{cm}}^{2})$$

where Q_T_ is the amount of RSV (mg or μg) entrapped in the tissue and A is the area available for penetration (0.636 cm^2^). The accumulation (A_c_) was then calculated by the relationship:

(4) $$\mathrm{Ac}=\frac{\mathrm{De}}{\mathrm{Cd}}\;(\mathrm{cm})$$

where C_d_ is the starting RSV concentration loaded into the donor compartment [34,35]. The Origin 8.5 software was used for mathematical data processing. Results are expressed as means ± standard error (repetition of 6 experiments).

#### 2.2.2. Preparation and Ex Vivo Evaluation of RSV-Loaded Mini-Tablets

##### Preparation of RSV-Loaded Pharmaceutical Powders by Spray Drying

To prepare RSV-loaded pharmaceutical powders using the spray drying technique, 5% (*w*/*v*) solutions that referred to the whole components in ethanol were prepared.

2.5 g of the excipients and RSV in the appropriate ratios (Table 1) were dissolved in ethanol (50 mL). In order to obtain the pharmaceutical powders, a BUCH MINI Spray Dryer I B-290 equipped with an inert B-295 loop was used, setting the following parameters: an inlet temperature of 110°C, a solution flow of 100 mL/h, a nitrogen aspiration of 100% and a cooling temperature (inert loop) of −20°C. Firstly, the instrument was equilibrated with 30 mL of fresh ethanol, and subsequently the prepared samples were processed. Afterwards, the instrument was cleaned with an additional 30 mL of fresh ethanol. Finally, the obtained powder was recovered.

##### Characterization of the Pharmaceutical Powders: Yield Percentage, Drug Loading Percentage (DL%) and Loading Efficacy Percentage (LE%)

Powders obtained by the spray drying technique were collected and immediately carefully weighed in order to calculate the yield as follows:

(5) $$\mathrm{Yield}\%=\frac{\mathrm{obtained}\;\mathrm{powder}\;\left(g\right)}{\mathrm{starting}\;\mathrm{components}\;\mathrm{amount}\;\left(g\right)}\times 100$$

Subsequently, to determine the amount of RSV in the samples as well as the powders’ homogeneity, randomly selected aliquots (5 mg) were accurately weighted, dissolved in methanol in a 10 mL flask and brought to volume with methanol. The resulting clear solutions were appropriately diluted, filtered through a 0.22 μm nylon membrane and immediately analyzed by HPLC, as described above. The drug loading percentage (DL%) and the loading efficacy percentage (LE%) were then calculated as follow:

(6) $$\mathrm{DL}\%=\frac{\mathrm{RSV}\;\left(\mathrm{mg}\right)}{\mathrm{RSV}-\mathrm{loaded}\;\mathrm{powder}\;\left(\mathrm{mg}\right)}\times 100\;\mathrm{LE}\%=\frac{\mathrm{real}\;\mathrm{RSV}\;\mathrm{amount}\;\left(\mathrm{mg}\right)}{\mathrm{theoretical}\;\mathrm{RSV}\;\mathrm{amount}\;\left(\mathrm{mg}\right)}\times 100$$

Each analysis was performed in triplicate and the results are reported as means ± standard error.

##### Preparation of RSV-Loaded Sublingual Mini-Tablets

In order to obtain standardized samples in terms of weight, volume and amount of RSV, the powders produced by spray drying were compressed in order to obtain sublingual tablets. Randomly selected aliquots of each powder were carefully weighed and directly compressed (6 tons) by using a hydraulic, single-die tableting machine (Perkin Elmer IR Accessory, Waltham, MA, USA), two flat-faced punches and a die [36]. To assess the reproducibility of the preparation method and tablets’ (volume 0.2 cm^3^) uniformity in terms of weight, 6 tablets for each batch of powder were evaluated (analytical balance, Mettler Toledo AE240, Columbus, OH, USA). Results are expressed as means ± standard error.

##### Ex Vivo Permeation Studies by Administering RSV-Loaded Sublingual Mini-Tablets

The ex vivo permeation studies by administering RSV-loaded sublingual mini-tablets were carried out analogously as described in paragraph 2.2.2. A sublingual mini-tablet was loaded into the donor compartment and soaked with 300 μL of citrate buffer (pH 5.5), while 15 mL of 3% β-CD (*w*/*v*) citrate buffer was used as acceptor fluid. At the end of each permeation experiment the donor medium was withdrawn, centrifuged, appropriately diluted in methanol and analyzed by HPLC (as described above) to determine the actual RSV concentration in the donor chamber. These data were employed as correct Cd values to calculate the Kp and Ac parameters. Moreover, at the end of experiments each mini-tablet was subjected to visual and tactile inspections in order to verify its integrity. Finally, the amount of RSV entrapped in the sublingual tissue was extracted and quantified as described above. The previously described biopharmaceutical parameters were calculated as reported above. Results are expressed as means of six experiments ± standard error.

##### Preparation and Characterization of CPE-Loaded Sublingual Mini-Tablets

The compositions indicated as RSV-B and RSV-C were chosen to include some selected CPEs (lysine hydrochloride, urea or menthol—ranging from 1 to 3% *w*/*v*), consequently reducing the PVP K90 amount necessary to maintain RSV–excipients ratios, as reported in Table 2. However, as LYS is insoluble in ethanol, to prepare the solution containing this CPE it was necessary to first dissolve LYS in 1.5 mL of bidistilled water and then add the clear solution to the ethanol solution of the other components to avoid LYS precipitation.

Powders obtained were collected and immediately weighed to evaluate the yield percentage and the loaded amount of RSV by HPLC analysis as described above. Finally, the prepared CPE-loaded powders were directly compressed as already reported and the obtained CPE-loaded sublingual mini-tablets were used to perform the ex vivo permeation studies.

#### 2.2.3. Characterization of RSV-B Powder and Sublingual Fast-Disintegrating Mini-Tablets

##### Scanning Electron Microscopy (SEM) Analysis

SEM measurements were carried out using a Zeiss EVO MA10 (Carl Zeiss Microscopy GmbH, Jena, Germany) scanning electron microscope, equipped with an Everhart–Thornley secondary electron detector (source of electrons was a lanthanum hexaboride LaB6 cathode). The accelerating voltage, 20 keV, and the probe voltage were 10 pA. The scanning electron microphotographs were acquired in an ultra-vacuum condition (HV, about 10^−7^ mbar) and at various magnification values. RSV-B powder was loaded on an aluminum stub and then coated with an ultrathin layer of gold (thickness about 2 nm) with an AGAR Sputter Coater-type system to increase the surface electrical conductivity.

##### Fourier Transform Infrared Spectroscopic in Attenuated Total Reflectance Mode (FTIR-ATR mode) Evaluations

FTIR-ATR mode spectra were recorded on a Fourier transform infrared spectrometer (Nicoret iS5, Thermo Scientific™, Waltham, MA, USA) equipped with a ZnSe ATR unit ID7 (Thermo Scientific™, USA) for surface analysis. Spectra were collected by the accumulation of 32 scans in the 4000-500 cm^−1^ range and rationed to the appropriate background spectra. Both pure RSV as well as the PVP K90 and RSV-B formulation were analyzed in order to evaluate any interaction.

##### X-ray Diffraction (XRD) Analysis

Pure RSV and the RSV-B formulation were evaluated by an X-ray diffractometer suitable for powders (D-8 Focus, Brucker, USA). The diffraction angles were scanned from 5° to 60° in θ/2θ at 2°/min, 40 kV voltage and 30 mA current at room temperature. XRD analyses were performed immediately after RSV-B powder preparation as well as after six months of storage at room temperature in the dark.

##### Disintegration Studies

In order to evaluate the disintegration time (DT) of the RSV-B mini-tablets, two different tests were performed.

- In Vitro Disintegration Test

To determine the DT a USP disintegration tester was used. The disintegration medium consists of 900 mL of distilled water heated at 37 ± 0.5 °C and stirred at 100 rpm [16]. The time required to observe the complete disintegration of each mini-tablet was recorded in seconds. Results are expressed as means of 6 experiments ± standard error.

- In Vitro Disintegration Visual Test

To better simulate the in vivo conditions a visual test was conducted. A mini-tablet was inserted into a glass crucible and soaked with 1 mL of citrate buffer solution (pH 5.5) prewarmed at 37 ± 0.5 °C. During the experiment both multiple photographs and short videos (normal speed or slow motion) were collected in order to visually assess the complete disintegration. The experiment was repeated for 6 randomly selected mini-tablets.

- In Vitro Dissolution Test

Dissolution tests were performed to evaluate the amount of RSV released over time. The tests were performed on 6 randomly selected RSV-B mini-tablets according to USP procedure using a USP apparatus type II method at a paddle speed of 50 rpm [16]. The dissolution medium consisted of 500 mL of citrate buffer (pH 5.5) at 37 ± 0.5 °C. At scheduled time intervals (2, 5, 8, 10, 15, 20 and 25 min) samples (1 mL) were withdrawn and replaced with fresh citrate buffer. The collected samples were centrifuged and the RSV amount was determined by UV-Vis analysis, as described above.

#### 2.2.4. Data Analysis

Data were expressed as means ± SE. All differences were statistically evaluated by the Student’s t-test with the minimum levels of significance, with p < 0.05.

## 3. Results and Discussion

### 3.1. Evaluation of CPEs to Promote RSV Sublingual Permeation

Considering the interesting broad-spectrum activities of RSV as well as its disadvantageous physicochemical properties, it is common to propose the use of alternative routes of administration aimed at overcoming the existing limits. In this context, the use of the sublingual route should show promising results. However, it is necessary to first evaluate the aptitude of RSV to cross the selected epithelial tissue by itself, as penetration and subsequent permeation phenomena are indispensable to ensure systemic effects. The main strategy to assess the permeability of an active ingredient through a biological membrane/tissue involves the use of a two-compartment open model (e.g., Franz-type diffusion cells) accompanied by a membrane that could be synthetic and accurately designed to simulate the biological tissue or could be a biological specimen [34,37,38,39]. Moreover, when evaluating the permeability of poorly water-soluble molecules, it is crucial to ameliorate their solubility into the donor compartment to increase the concentration gradient, which is indispensable in establishing the permeation phenomenon. In addition, it is also crucial to select an acceptor fluid in which the drug is freely soluble to be sure that any limitation in terms of permeation is just due to the characteristics of the drug instead of restrictions by the saturation of the acceptor medium. Previous studies confirmed the usefulness of citrate buffer solution (pH 5.5) to stabilize and of β-cyclodextrins (3% *w/w*, β-CD) to promote solubility of RSV [4]. Nevertheless, the β-CD solution in citrate buffer tends to give rise to precipitation phenomena if not subjected to constant agitation and, consequently, it was not used as donor fluid as it led to a variation in the effective area of the mucosa available to permeation, thus producing misleading results.

**Figure 1:**
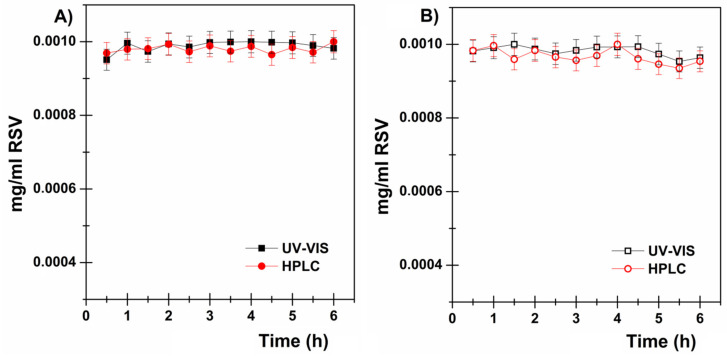
RSV stability studies on (**A**) the citrate buffer (pH 5.5) and PEG_200_ mixture (80:20 *v*/*v*) and (**B**) also in presence of NAC (RSV: NAC = 1:2). RSV was quantified by UV-Vis (black) and HPLC (red) analyses. Results are presented as means ± SE (*n* = 3).

Therefore, in order to find an effective strategy with which to load high RSV concentrations into the donor chamber, the following parameters were considered: RSV stability is enhanced at acidic pH values; RSV is extremely poorly water-soluble (logP = 3.1; water solubility = 0.05 mg/mL); and RSV is extremely soluble in polyethylene glycol (e.g., PEG_400_ solubility = 373.85 mg/mL) [13,14]. In particular, the mixture of citrate buffer solution (pH 5.5) + PEG_200_ (80:20 *v*/*v*) was proposed, as it was able to easily dissolve high RSV concentrations (1 mg/mL). To evaluate the usefulness of the proposed mixture, RSV stability studies were performed both with and without NAC, which is used as a preservative agent for polyphenols [40]. Stability studies were performed in the dark for 6 h at 37 ± 0.5 °C and the amount of RSV in each sample was quantified by both UV-Vis as well as HPLC analyses. As reported in Figure 1, RSV remains stable in both cases for the whole time considered and, consequently, the mixture without NAC was selected for further ex vivo evaluations.

Once selected, both the donor and the acceptor fluids (citrate buffer + PEG_200_ mixture and the 3% β-CD dispersion in citrate buffer, respectively) ex vivo permeation studies in the absence and presence of six selected CPEs were performed. In particular, the following CPEs belonging to different chemical classes were used:

- Sodium dodecylsulfate (SDS). It is a widely used anionic surfactant consisting of a hydrophobic tail (C12) linked to a hydrophilic sulfate group. It appears as a white crystalline powder, is quite soluble in water and ethanol and is often used in the cosmetic and pharmaceutical fields to promote solubility as well as absorption of actives through epithelial membranes, e.g., skin and gastrointestinal mucosa. The permeation enhancer effects are attributable to the alteration of the ordered state of the extracellular lipids by solubilization [23,24,41];
- Sodium dehydrocolate (SDC). It belongs to the class of biliary salts/acids which are defined as amphipathic ionic biosurfactants with a steroid structure, as they are synthesized in the liver from cholesterol. Thanks to its high biocompatibility, it could be widely used as a permeation enhancer through skin buccal, nasal, lung and intestinal tissues. Moreover, it exerts chemical and enzymatic stabilization of drugs. The absorption enhancement effect is due to the extraction of membrane proteins, interaction with the lipid component of the membranes and the formation of inverse micelles which reversibly increase the fluidity of the apical and basolateral membranes, thus facilitating the passage of drugs [22,42];
- Transcutol^®^ (T). It is also known as diethylene glycol monoethyl ether. It is a clear liquid characterized by low viscosity, a stability between pH 4-9 and a pleasant odor. It acts as a permeation enhancer by improving drug solubility inside the membranes (alteration of the partition coefficient) rather than directly increasing the drug diffusivity [43];
- Lysine hydrochloride (LYS). It is a cationic amino acid that belongs to the twenty essential amino acids and, consequently, it is biocompatible, safe and non-toxic. Its chemical permeation enhancement effect is mainly due to the establishment of ionic interactions with the charged groups of the mucosal membrane, thus increasing the diffusion process. Furthermore, lysine could benefit from the amino acid transporters and consequently direct active substances through the epithelial layers [26,44];
- Urea (U). It is a biocompatible organic compound that appears as a colorless crystalline solid. Numerous studies have depicted its permeation enhancement ability because of its highly moisturizing power (ability to recall and retain water). Moreover, urea is also able to increase the fluidity of the phospholipid bilayer while maintaining the integrity of the membrane protein domains [27];
- Menthol (M). It belongs to the terpenes, which are reported as permeation promoters obtained from natural sources and widely included on the Food and Drug Administration (FDA) list of safe agents. The permeation enhancement power of terpenes is mainly linked to their chemical structure and their physicochemical properties. In particular, menthol is able to increase the interaction with the non-polar membranes and it is also useful as a flavoring agent, thus increasing patients’ compliance [45].

**Figure 2:**
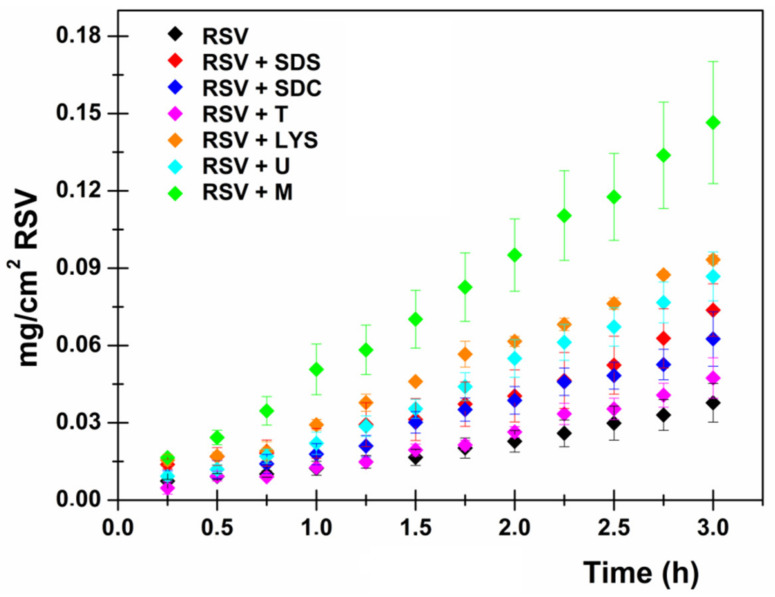
RSV permeation profiles in the absence and in the presence of CPEs. RSV (mg/cm^2^) permeated as a function of incubation time (h) after administration of RSV solution 1 mg/mL (black) and RSV solution 1 mg/mL + 0.2 mg/mL of sodium dodecylsulfate (red), sodium dehydrocolate (blue), Transcutol^®^ (pink), lysine hydrochloride (orange), urea (light blue) and menthol (green). Results are presented as means ± SE (*n* = 6).

**Figure 3:**
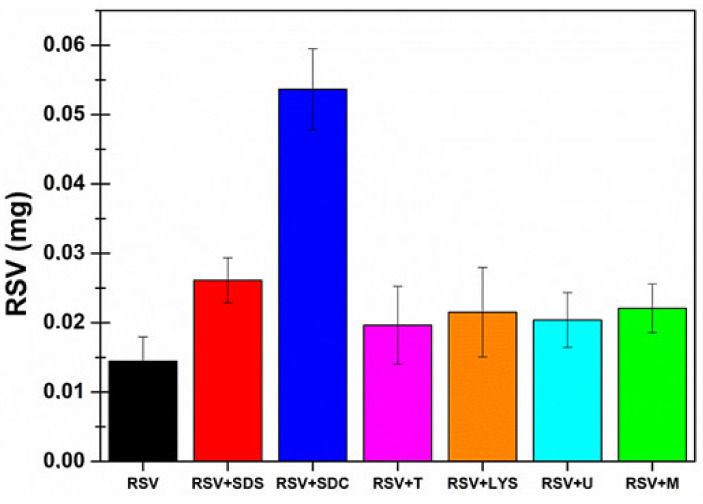
Amount (mg) of RSV accumulated in the sublingual tissue after 3 h by administering RSV solution 1 mg/mL (black) and RSV solution 1 mg/mL + 0.2 mg/mL of sodium dodecylsulfate (red), sodium dehydrocolate (blue), Transcutol^®^ (pink), lysine hydrochloride (orange), urea (light blue) and menthol (green). Results are presented as means ± SE (*n* = 6).

For each set of the permeation experiments the RSV concentration was kept constant (1 mg/mL), as was the RSV:CPE ratio (5:1). Drug permeation profiles are reported in Figure 2, while Figure 3 shows the amount of RSV entrapped in the sublingual tissue at the end of the permeation experiments. To singularly observe the permeation profiles please check the Supplementary Materials (from Figure S1 to Figure S7). Finally, Table 3 highlights the calculated biopharmaceutical parameters.

As is observable, each of the selected CPEs is capable of promoting RSV permeation through the sublingual mucosa as well as its accumulation in the tissue. As reported, the enhancement in terms of drug flux follows the trend RSV < RSV+T < RSV+SDC < RSV+SDS < RSV+U < RSV+LYS < RSV+M, while the promotion of tissue accumulation follows the trend RSV < RSV+T < RSV+U < RSV+LYS < RSV+M < RSV+SDS < RSV+SDC. These data generally confirm that Transcuol^®^ exhibits the lowest permeation/penetration enhancement activity. Moreover, it is likely noticeable that SDS and SDC are better at promoting tissue accumulation rather than drug permeation. However, it is generally observable that the flux increase is accompanied by a variation/reduction in the calculated t_lag_ value.

To briefly summarize, the best permeation enhancement activity was exerted by menthol, lysine and urea, which increase RSV flux through the mucosa up to 3.5, 2.4 and 2.3 times, respectively, which correspond to an increase of up to 247.4%, 136.0% and 134.5% in terms of Kp value. These CPEs were then selected to be added to the most promising spray-dried compositions as they represented the best lipophilic CPE (menthol), the best hydrophilic pH-dependent CPE (lysine) and the best hydrophilic pH-independent CPE (urea).

### 3.2. Preparation and Characterization of RSV-Loaded Mini-Tablets by Spray Drying Technique

At this point, the second aim of the present work was to develop RSV-loaded spray-dried powders suitable for preparing mini-tablets for sublingual application. This goal is based on the potential effectiveness of innovative sublingual DDSs capable of improving RSV solubility, stability, administrability and consequently, permeability. Sublingual mini-tablets were selected as a suitable innovative dosage form as they were optimal vehicles in which to load a large amount of drug. The key factor in producing appropriate sublingual solid formulations is to start from a homogeneous powder, and thus the spray drying technique was chosen to prepare four different powders in terms of composition/excipients ratios, as reported in the Materials and Methods section. In particular, the prepared powders (named RSV-A, RSV-B, RSV-C and RSV-D) were composed by a fixed RSV amount (15% *w*/*w*) and different ratios of the following excipients: PVP K90, PEG_200_, sorbitol and propylene glycol. The main component of the proposed formulations is polyvinylpyrrolidone K90 (PVP K90). It is a synthetic polymer consisting of 1-vinyl-2-pyrrolidone monomers, soluble in water, alcohols and other polar solvents. Thanks to its versatility, PVP K90 is a widely used excipient in the pharmaceutical, food and cosmetic industries. Indeed, PVP K90 is a biodegradable, stable and non-toxic polymer, able to interact with both hydrophilic and lipophilic molecules and is therefore useful for the development of conventional as well as innovative DDSs. PVP K90 is also used to improve the solubility and enhance the dissolution rate and thus the bioavailability of poorly water-soluble actives. Furthermore, it increases the physical stability of amorphous drug forms by preventing crystallization. Additionally, PVP K90 is often used to prepare mucosal DDSs due to its mucoadhesive properties [46,47]. To promote RSV solubilization into the buccal environment, PEG_200_ was chosen as the secondary polymer. PEGs are characterized by high structural flexibility (without any steric hindrance), biocompatibility and high hydration capability. In addition, they are biodegradable polymers with a well-established safety profile, being approved by the FDA. In particular, PEG_200_ was chosen due to its solubilizing/stabilizing and taste-masking properties as well as its ability to act as a promoter of drug bioavailability [48,49]. In designing fast-disintegrating formulations the role of the disintegrating agent is significant, which, in this work, was identified as sorbitol. Sorbitol is the alditol of glucose. It is a hydrophilic molecule capable of attracting and retaining water, thus determining the fast dissolution of formulations on the application site and promoting in situ high drug concentration. It is widely used in the food industry for its sweetening power (about 60% of sucrose) and its role as a stabilizer and leavening agent. Its interesting properties also make it very useful in the pharmaceutical field. In this context, sorbitol proved to be an advantageous excipient in the production of sublingual fast-disintegrating tablets, being capable of rapidly disaggregating into the small salivary volumes and contextually showing pleasant organoleptic characteristics [50]. Finally, only in the RSV-D formulation was propylene glycol added. It is an odorless, colorless and viscous liquid, characterized by a sweetish taste, hygroscopicity and miscibility with different solvents. It is widely used in the food, cosmetic and pharmaceutical fields as an additive, plasticizer, humectant and solubilizer. In this work it was selected as it can act as an excellent cosolvent to increase RSV solubility, thus synergizing with the effect of PEG_200_ [51].

To summarize: the RSV-A formulation could be considered the basic one, RSV-B is characterized by the highest amount of sorbitol, RSV-C comprised the largest amount of PEG_200_ and RSV-D is the formulation that includes propylene glycol as an excipient. The different compositions resulted in different spray-dried powder appearances. In particular, RSV-A (see Supplementary Materials: Figure S8, panel A) and RSV-B had soft and light powders while RSV-C (see Supplementary Materials: Figure S8, panel B) and RSV-D lead to very fine and dense powders.

In order to evaluate the suitability of the spray drying technique and verify the employment of the correct procedure parameters, each obtained powder was subjected to yield %, DL% and LE% evaluations. The results are reported in Table 4 and highlight the successful application of the spray drying method. Indeed, the yield % of around 70% *w/w* is a really high value as, according to the literature, a significant disadvantage of the spray drying process concerns a non-optimal atomization yield (20–70%) due to the product loss in the walls of the drying chamber and the low ability of the cyclone in separating the fine particles [31]. Moreover, the obtained DL% and LE% values confirmed the homogeneity of the prepared powders and the reproducibility of the preparation method. The data obtained by HPLC-DAD analysis also proved that RSV exposure to the high operating temperature (110 °C) did not involve any alteration and/or degradation. In fact, the RSV retention time and UV-Vis spectrum were identical to the RSV standard. Neither additional chromatographic peaks nor alterations in the shape of the UV spectrum at wavelengths between 200 and 700 nm were observed during quantification analyses [52]. This evidence ruled out any doubt in the maintenance of RSV stability during the production process.

The prepared powders were then directly compressed in order to produce sublingual mini-tablets (see Supplementary Materials: Figure S9) that were homogeneous in terms of weight and RSV content (Table 5), thus confirming the reproducibility of the direct compression method.

**Figure 4:**
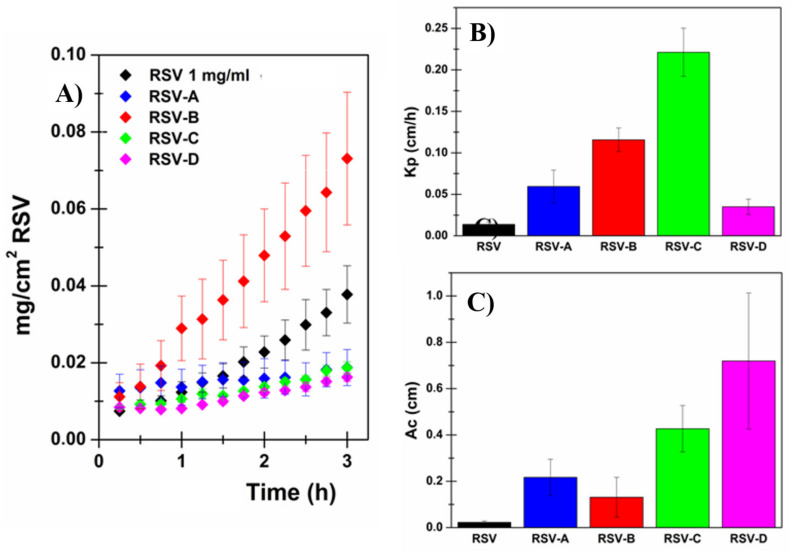
(**A**) RSV permeation profiles: RSV (mg/cm^2^) permeated as a function of incubation time (h). (**B**) Kp and (**C**) Ac parameters, calculated after administration of RSV solution 1 mg/mL (black), RSV-A mini-tablet (blue), RSV-B mini-tablet (red), RSV-C mini-tablet (green) and RSV-D mini-tablet (pink). Results are presented as means ± SE (*n* = 6).

The prepared mini-tablets were used to evaluate RSV permeation when embedded into the proposed formulation by using Franz-type vertical diffusion cells and porcine sublingual mucosae as membrane models. Each mini-tablet was placed into the donor chamber and soaked with 300 μl of citrate buffer solution (pH 5.5). To better understand the obtained results (Figure 4 and Table 6) it is important to consider the extremely low water solubility of RSV. Indeed, to correctly calculate the Kp and Ac parameters, the actual RSV concentration in the donor chamber was evaluated and considered at the end of each permeation experiment. Moreover, after permeation studies, the mini-tablets were subjected to visual and tactile inspections. As expected, the variations in terms of composition resulted in different behaviors. RSV-A mini-tablets absorbed water from the surrounding environment and became rubbery, similar to chewing gum; RSV-B mini-tablets completely disintegrated once in contact with the donor media (as they contain the highest amount of sorbitol); and RSV-C as well as RSV-D mini-tablets were only partially disintegrated, and some solid residues were extremely crumbly to the touch.

These observations are in agreement with the experimental data. Indeed, the RSV-B formulation, which is the only one that completely disintegrates, allowed the highest RSV concentration into the donor compartment (192.44 ± 37.40 μg/mL). Moreover, it should be considered that this concentration value is extremely high when compared to the reported water solubility of RSV (50 μg/mL), thus confirming the ability of the proposed formulation to enhance drug solubility. Even more relevant is that RSV-B dosage form resulted in the highest observed flux value (22.26 ± 4.78 μg/cm^2^∙h^−1^). This result is of crucial importance, especially when compared to the flux obtained by administering 1 mg/mL RSV solution: the RSV solution, despite having a concentration five-fold higher than the RSV-B formulation, produces a drug flux 1.6-fold lower.

Consistent again with what was observed at the end of the experiments, RSV-C and RSV-D exhibited similar flux values while RSV-A had the lowest flux (1.95 ± 0.64 μg/cm^2^ ∙ h^−1^). However, for a more complete evaluation, Kp and Ac parameters (which are concentration independent) should be considered as the RSV concentration in the donor chamber is strictly correlated to the results obtained. By observing these values, it was evident that all four proposed formulations were able to act both as permeation (increased Kp) and penetration (increased Ac) enhancers for RSV. Generally, RSV-A, RSV-B, RSV-C and RSV-D compositions increased the Kp value up to 4.3 (332.0%), 8.4 (740.6%), 16.1 (1507.2%) and 2.7 (154.5%) times respectively when compared to RSV solution by itself. These results are highly significant and particularly satisfactory, especially when compared to those obtained by simply employing the CPEs (the best CPE was menthol, which enhances RSV permeability up to 3.5 times (247.4%)). In particular, RSV-B and RSV-C resulted in the highest Kp values (0.11566 ± 0.01422 cm/h and 0.22115 ± 0.02894 cm/h, respectively, vs. 0.01376 ± 0.00311 cm/h for RSV solution).

To summarize: RSV-B and RSV-C appeared to be the best proposed formulations, and they were thus selected to be loaded with the best CPEs (menthol, lysine and urea).

Five different CPE-containing compositions were produced by spray drying and, after characterization, were compressed into mini-tablets. The first significant difference between the CPE-loaded powders and the corresponding CPE-free formulations was related to the appearance. Indeed, the presence of the CPE, even a small amount (1–3% *w*/*w*), completely modified the obtained powders, which were more cohesive and compact. The other evaluated characteristics for both the powders and the mini-tablets are reported in Table 7 and confirmed the suitability of the spray drying process and the reproducibility of the tablet preparation method.

**Figure 5:**
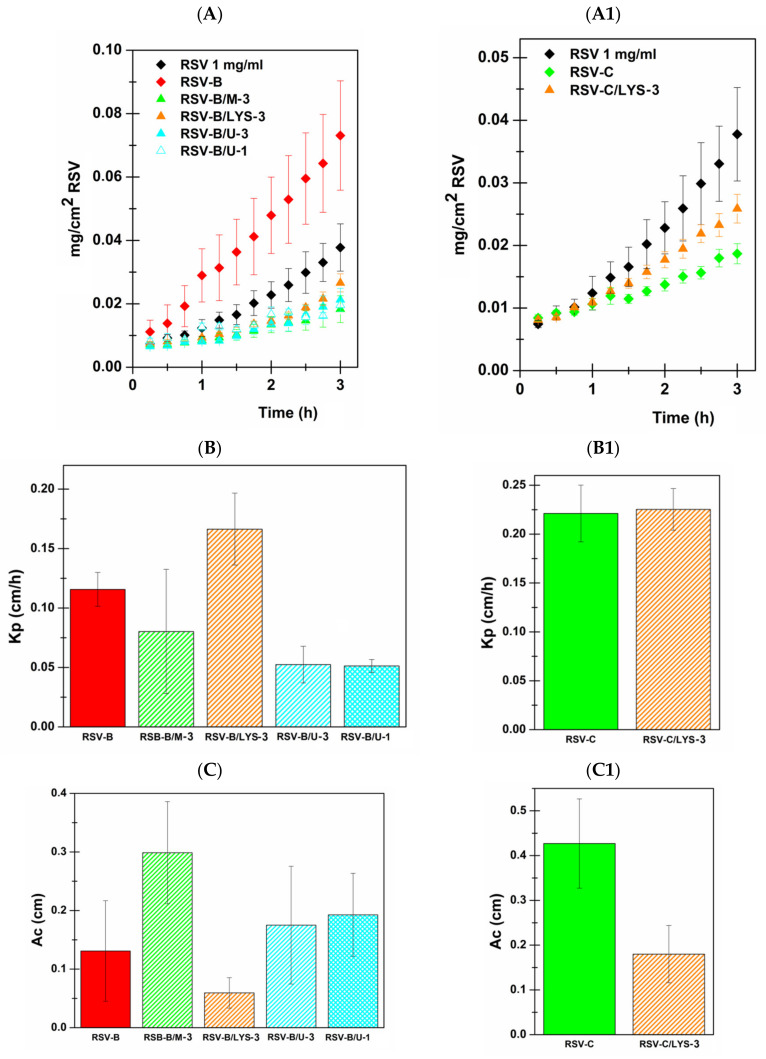
(**A**,**A1**): Amount of RSV (mg/cm^2^) permeated from mini-tablets as a function of time (h). (**B**,**B1**): Kp; (**C**,**C1**) Ac parameters calculated after administration of RSV solution 1 mg/mL (black), RSV-B mini tablet vs. CPE-loaded RSV-B (**A**–**C**) and RSV-C mini tablet vs CPE-loaded RSV-C (**A1**–**C1**). Results are presented as means ± SE (*n* = 6).

It is not surprising that the observed variations in terms of powders’ appearances resulted in different behaviors in terms of disintegration and RSV permeation. In particular, the CPE-loaded mini-tablets underwent only partial disintegration after permeation tests, and the solid residues were extremely crumbly to the touch. This generally resulted in a lower RSV concentration value and thus lower or at least similar flux, Kp and Ac values (Figure 5 and Table 8) than those of the corresponding CPE-free formulation. Moreover, the lower observed efficacy of the selected CPE when inserted into the formulations, rather than when used as a solution together with RSV, could be due to the following considerations: menthol is a hydrophobic molecule insoluble in citrate buffer solution, and thus in these experimental conditions it is probably not solubilized and, consequently, it could not act; unexpectedly, lysine fails to be effective, perhaps due to its pH-dependent dissociation. The contribution of the PEG_200_ used in the previous experiments probably increased the undissociated fraction available to act and favored the solubility of menthol. Finally, the unsatisfactory results obtained from the urea-loaded mini-tablets chould be due to the previously highlighted differences in terms of powder appearance. The cohesion as well as the incomplete disintegration of the urea-loaded powders/mini-tablets could also be due to the high moisturizing power of this permeation enhancer. The drying process of the component’s mixture could potentially have determined interactions between these and urea, which should be deepened with further studies, or some excipients should simply be replaced.

These results, unfortunately, preclude the possibility of merging the CPE-based approach and the innovative spray drying method proposed to enhance RSV permeation through the sublingual tissue. In any case, it is important to point out that all the proposed CPE-loaded formulations were able to enhance RSV sublingual permeability. In particular, RSV-B/M-3, RSV-B/LYS-3, RSV-B/U-3, RSV-B/U-1 and RSV-C/LYS-3 increased RSV permeability (Kp enhancement) up to 5.8 (483.1%), 12.1 (1108.9%), 3.8 (280.8%), 3.7 (272.6%) and 16.4 (1537.4%) times, respectively.

**Figure 6:**
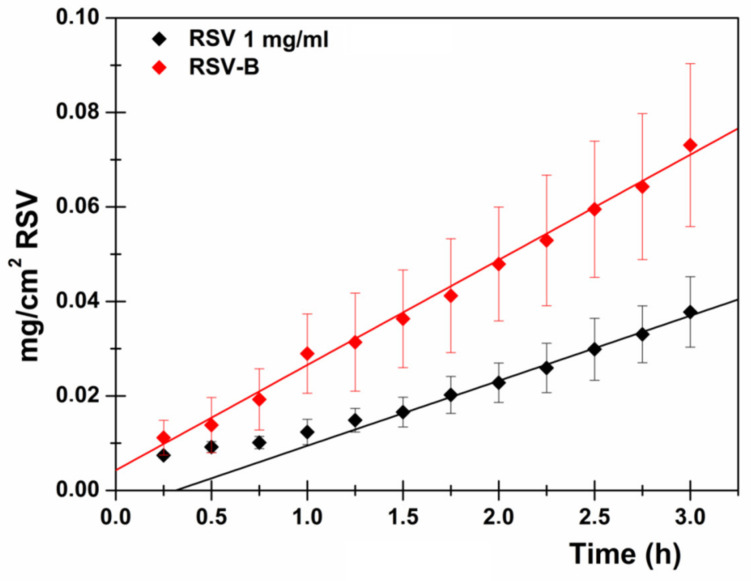
RSV (mg/cm^2^) permeated as a function of time (h) after administration of 1 mg/mL RSV solution (black) and RSV-B mini-tablet (red).

To summarize, the RSV-B composition resulted in the best mini-tablet formulation due to the following considerations: the tablet appears totally disintegrated at the end of the permeation experiments and additionally the disintegration process begins instantly as soon as the formulation is put into contact with the donor fluid; the RSV-B formulation allows high RSV concentration in the donor compartment, thus contrasting the well-known poor water solubility of RSV and consequently contributing to the obtainment of an effective concentration gradient; the observed drug flux is higher than that observed by administering RSV solution 1 mg/mL (Figure 6), although drug concentration in the donor compartment is about 1/5 (0.192 mg/mL for the RSV-B formulation vs. 1 mg/mL for the RSV solution); and the RSV-B mini-tablets promote immediate drug absorption and immediate establishment of the steady-state equilibrium (no lag time). All these factors might contribute to determining high patient compliance and potentially therapeutic success. Indeed, after in vivo administration high blood concentrations of RSV could be achieved as the mini-tablet disintegration could lead to a sublingual highly concentrated RSV solution which is in close contact with a great surface of absorption (tongue ventral surface together with floor of the mouth are about 26.5 cm^2^) [17].

### 3.3. Characterization of RSV-B Powder and Mini-Tablets

As already highlighted, the RSV-B composition was the most promising one both in terms of enhancement of RSV solubility and thus flux and permeability, as well as in terms of suitability as a fast-disintegrating sublingual formulation. In view of the obtained promising results, the selected composition was then subjected to further evaluations.

In particular, the RSV-B powder was investigated by SEM, FTIR in ATR mode and XRD analyses.

SEM images were recorded to verify sample homogeneity as well as the size and the morphology of the spray-dried microparticles which make up the obtained powder.

**Figure 7:**
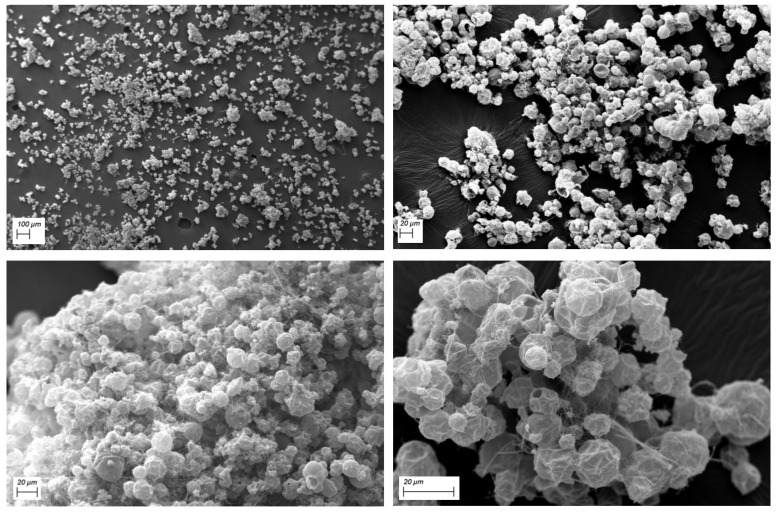
Morphology of RSV-B spray-dried powder evaluated by scanning electron microscopy (SEM) at different magnifications in order to verify microparticles’ diameter and sample uniformity.

Figure 7 shows different images of the RSV-B powder, recorded at various magnifications. As is observable, the analyzed sample is homogeneous with microparticles ranging from 15 to 20 μm and linked by a web-like structure, probably due to the presence of PVP K90.

**Figure 8:**
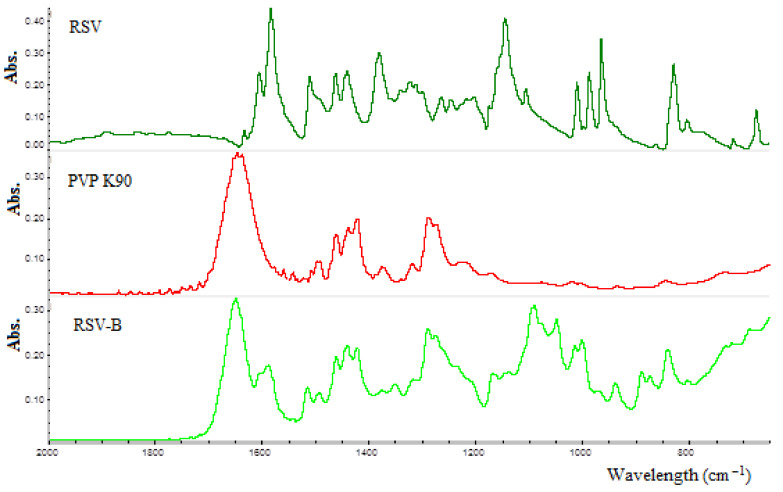
ATR-FTIR spectra of pure RSV (black), PVP K90 (red) and RSV-B powder (green).

Furthermore, FTIR spectra were collected in ATR mode to evaluate any drug–excipients interaction. As shown in Figure 8, the presence of PVP K90 allows for the hiding of RSV peaks. Analyzing the recorded spectra in the range from 1550 to 1620 cm^−1^, it is possible to notice the presence of RSV peaks without any chemical shift. Moreover, at 1650 cm^−1^ a mathematical sum of RSV and PVP K90 peaks further confirmed that no chemical interactions occur between the natural compound and the employed polymer.

**Figure 9:**
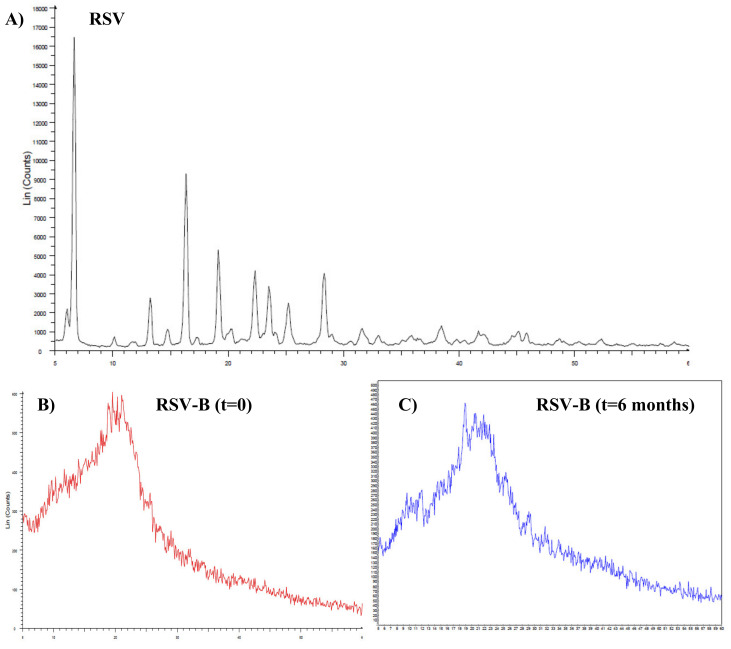
Diffractograms of (**A**) pure RSV, (**B**) RSV-B immediately after preparation and (**C**) RSV-B after a 6-month storage period.

Finally, the last crucial characterization performed on the RSV-B-spray-dried powder was aimed at the evaluation of RSV physical state (crystalline or amorphous) by XRD analysis. The obtainment of the drug in its amorphous form is a key point aimed at improving drug solubility and consequently permeability, particularly for poorly water-soluble molecules such as RSV. Additionally, from a thermodynamic point of view, an amorphous form is not stable and could easily evolve again into the crystalline form, so it is also crucial to assess its stability over time. In Figure 9 the obtained diffractograms for both RSV and RSV-B formulation are reported. As is observable, panel A is characterized by the peaks of the starting employed crystalline, RSV, while panel B is characterized by the absence of the previously observed peaks, being of RSV-B powder. This is due to the complete amorphization of RSV when spray-dried together with the selected excipients. Furthermore, panel C shows that after 6 months of storage of the prepared powder (at room temperature, in the dark) the loaded RSV remains in the amorphous form. This is probably due to the efficacy of PVP K90 in preventing drug crystallization by enhancing the physical stability of the amorphous form [46,47]. These results are particularly interesting and satisfactory, as well as being perfectly coherent with the observed enhancement of RSV solubility for the RSV-B formulation.

Moreover, the prepared RSV-B mini-tablets were subjected to disintegration and dissolution studies.

**Figure 10:**
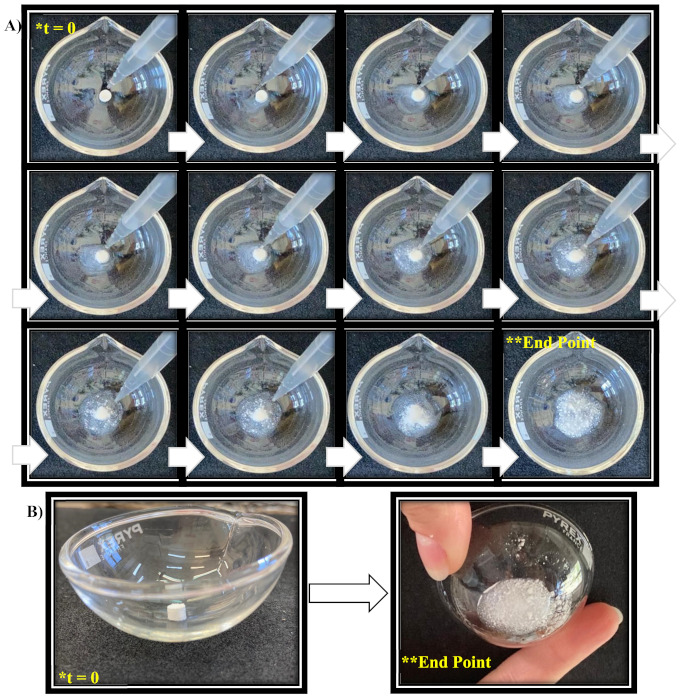
In vitro disintegration visual test: (**A**) sequence of photographs; (**B**) highlights of the starting and end points (for a more complete result please check the videos in the Supplementary Materials).

The disintegration time (DT) is a critical attribute to be optimized for the aim of preparing an effective fast-disintegrating sublingual formulation. Generally, it is reported that the observable DT, by using a disintegration apparatus, should be less than 30 sec for orally disintegrating tablets [29,53,54]. In this work, the DT was evaluated by two different methods. The first one, as the literature reports, consists in the use of a large disintegration medium volume (900 mL) at 37 ± 0.5°C [16]. In these experimental conditions, the calculated DT was 4.06 ± 0.39 sec. Moreover, to permit an easy evaluation, a visual disintegration experiment was conducted by recording both photographs and videos of the disintegration process. This type of test was performed by inserting RSV-B mini-tablets into glass crucibles and soaking them with 1 mL of prewarmed (37 ± 0.5°C) citric buffer solution (pH 5.5). Indeed, the use of such a small volume is more representative as it mimics the in vivo conditions observable after sublingual administration (the mini-tablet will be in contact with a small salivary volume). This test confirms the previously obtained results in terms of DT (Figure 10). As the disintegration process was very fast it was quite difficult to attribute a time point (sec) to all the proposed pictures and, as a consequence, in the Supplementary Materials two videos are reported (normal speed and slow motion) to better illustrate the disintegration of the proposed RSV-B mini-tablets. However, it must be considered that the whole experiment required at least 8 s from the beginning of soaking to complete disintegration. Indeed, in any case the proposed formulation meets the criteria for a sublingual fast-disintegration dosage form (DT < 30 s).

**Figure 11:**
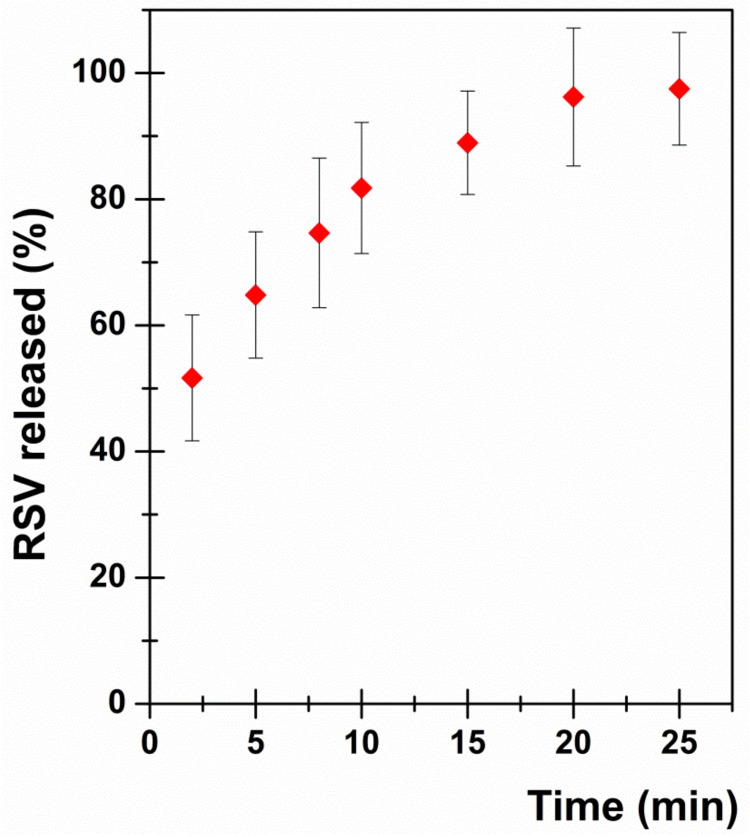
In vitro dissolution profile: RSV released (% of dose) as a function of incubation time (min). Results are presented as means ± SE (*n* = 6).

Another parameter to be evaluated, especially for poorly water-soluble molecules, is drug dissolution rate. The dissolution studies were performed according to the literature, which reports that the percentage of released drug from sublingual tablets must exceed 80.0% in 15 min [54,55]. Results are reported in Figure 11 as percentage of RSV dose released over time (min). As is observable, after 15 min 88.94 ± 8.19% of the RSV dose was released, thus confirming the goodness of the proposed formulation. Furthermore, the whole amount of RSV was released at the end of the dissolution tests (25 min).

To conclude, the proposed RSV-B mini-tablets should be suitable and effective as a sublingual fast-disintegrating dosage form as they meet all the quality criteria of a useful sublingual DDS:

- Ability to completely and quickly disintegrate to ensure good patient compliance (DT: 4.06 ± 0.39 s);
- Ability to quickly release the embedded drug thus ensuring the formation of a concentrated in situ solution (RSV released after 15 min: 88.94 ± 8.19%);
- Ability to promote drug solubility (RSV concentration in the donor chamber: 192.44 ± 37.40 μg/mL);
- Ability to ensure a high drug flux through the administration site in order to maximize its entrance into the bloodstream allowing systemic effects (RSV flux after RSV-B administration: 22.26 ± 4.78 μg/cm^2^∙h^−1^);
- Ability to promote an immediate absorption, allowing a fast achievement of the desired effects (lag time absence; 8.41-fold increased Kp value with respect to RSV solution 1 mg/mL).

## 4. Conclusions

In this work, the low aptitude of RSV to cross the sublingual mucosa was successfully overcome by two different strategies. On one hand, six different CPEs (SDS, SDC, Transcutol^®^, urea, lysine and menthol) were tested and they all were useful in promoting sublingual RSV permeation. In particular, menthol, lysine and urea were the best hydrophobic, hydrophilic pH-dependent and hydrophilic pH-independent CPEs, respectively. On the other hand, the spray drying technique was used as an innovative approach to prepare RSV-loaded powders suitable for direct compression in order to obtain sublingual mini-tablets. Four different compositions named RSV-A, RSV-B, RSV-C and RSV-D containing high amounts of RSV (15% *w/w*) were prepared by varying the excipients ratios (PVP K90, PEG_200_, sorbitol and propylene glycol). RSV was proven to be a good candidate for the spray drying process as the operating conditions do not compromise its stability. The obtained powders were homogenous and the spray drying atomization yield was particularly high (≈ 70%). Moreover, the obtained pharmaceutical powders allowed for the creation of uniform mini-tablets which were used for the ex vivo permeation evaluations. All the proposed formulations were able to enhance RSV sublingual permeation (increments in terms of Kp) and accumulation in the mucosal tissue (increments in terms of Ac). As data suggested, the RSV-B formulation emerged as the most promising one while the RSV-C composition allowed for the highest observed increment in terms of Kp. These chosen compositions were then added to the best CPEs, thus preparing CPE-loaded powders and mini-tablets. Unfortunately, these formulations gave unsatisfactory results both in terms of RSV solubility, drug flux and Kp values enhancement. These results thus preclude the possibility of converging the two proposed approaches (the chemical and the technological ones) to promote RSV sublingual permeation. As the RSV-B composition was the most promising one, it was further analyzed in order to demonstrate its usefulness as a fast-disintegrating sublingual formulation. In particular, SEM analysis highlighted the particle size homogeneity of the spray-dried powder (≈ 15-20 μm), FTIR spectra depicted that no interactions occur between RSV and PVP K90 and the XRD evaluation showed the complete and stable amorphization of the loaded RSV after 6 months of storage. Finally, its effectiveness as a fast-disintegrating sublingual dosage form was assessed by the evaluation of the disintegration time (4.06 ± 0.39 sec) and the percentage of RSV dose released after 15 min (88.94 ± 8.19%). The results obtained perfectly fit with the reference values reported in the literature.

## Figures and Tables

**Table 1:** Percentage composition of RSV-loaded pharmaceutical powders.

	PVP K90 (%)	PEG_200_ (%)	Sorbitol (%)	Propylene Glycol (%)	Trans-Resveratrol (%)
**RSV-A**	59	20	6	-	15
**RSV-B**	45	20	20	-	15
**RSV-C**	49	30	6	-	15
**RSV-D**	35	20	10	10	15

**Table 2:** Composition (as *w*/*w* percent) of RSV-loaded pharmaceutical powders that include the selected CPEs.

	PVP K90 (%)	PEG_200_ (%)	Sorbitol (%)	Trans-Resveratrol (%)	Lysine (%)	Menthol (%)	Urea (%)
**RSV-B/LYS-3**	45	20	20	15	3	-	-
**RSV-B/M-3**	45	20	20	15	-	3	-
**RSV-B/U-3**	45	20	20	15	-	-	3
**RSV-B/U-1**	45	20	20	15	-	-	1
**RSV-C/LYS-3**	49	30	6	15	3	-	-

**Table 3:** Extrapolated RSV biopharmaceutical parameters (Js, Kp, t_lag_, De and Ac) in the absence or in the presence of the selected CPEs. Values are presented as means ± SE (*n* = 6).

	Js (mg/cm^2^∙h^−1^) = K_p_ (cm/h) *	t_lag_ (min)	De (mg/cm^2^) = Ac (cm) *
**RSV**	0.01376 ± 0.00311	19 min	0.02271 ± 0.00554
**RSV+T**	0.01829 ± 0.00347	29 min	0.03086 ± 0.00883
**RSV+SDC**	0.02139 ± 0.00401	11 min	0.08435 ± 0.00922
**RSV+SDS**	0.02718 ± 0.00286	26 min	0.04108 ± 0.00510
**RSV+U**	0.03226 ± 0.00220	22 min	0.03203 ± 0.00622
**RSV+LYS**	0.03247 ± 0.00067	5 min	0.03384 ± 0.01013
**RSV+M**	0.04780 ± 0.00896	–	0.03472 ± 0.00553

*, J_s_ = K_p_ and De = Ac because Cd = 1 mg/mL.

**Table 4:** Characterization of the spray-dried powders in terms of yield %, DL% and LE% ± SE (*n* = 3).

Composition	Yield %	DL%	LE%
**RSV-A**	76.0	16.54 ± 1.06	110.29 ± 7.09
**RSV-B**	82.0	11.06 ± 0.67	73.71 ± 4.50
**RSV-C**	64.4	15.41 ± 0.44	102.73 ± 2.95
**RSV-D**	67.6	15.67 ± 0.33	102.44 ± 2.22

**Table 5:** RSV-loaded sublingual mini-tablets: weight and drug content ± SE (*n* = 6).

Formulation	Weight (mg)	RSV per Mini-Tablet (mg)
**RSV-A mini-tablet**	29.96 ± 0.38	4.96 ± 0.06
**RSV-B mini-tablet**	30.60 ± 0.47	3.28 ± 0.05
**RSV-C mini-tablet**	30.59 ± 0.12	4.71 ± 0.02
**RSV-D mini-tablet**	30.82 ± 0.40	4.74 ± 0.06

**Table 6:** Extrapolated RSV biopharmaceutical parameters (Js, Kp, t_lag_, De and Ac ± SE) and RSV concentration in the donor chamber after mini-tablets ex vivo evaluation.

Sample	J_s_ (μg/cm^2^∙h^−1^)	K_p_ (cm/h)	t_lag_ (min)	De (μg/cm^2^)	Ac (cm)	(RSV)_DONOR_ (μg/mL)
**RSV**	13.76 ± 3.11	0.01376 ± 0.00311	19	22.71 ± 5.54	0.02271 ± 0.00554	1000 (STD)
**RSV-A**	1.95 ± 0.64	0.05944 ± 0.01981	NO	7.12 ± 1.58	0.21702 ± 0.07829	32.74 ± 4.60
**RSV-B**	22.26 ± 4.78	0.11566 ± 0.01422	NO	25.19 ± 9.36	0.13087 ± 0.08584	192.44 ± 37.40
**RSV-C**	3.70 ± 0.35	0.22115 ± 0.02894	NO	7.13 ± 1.07	0.42688 ± 0.09983	16.71 ± 2.17
**RSV-D**	3.95 ± 0.55	0.03502 ± 0.00932	NO	81.18 ± 5.22	0.71939 ± 0.29361	112.85 ± 16.76

**Table 7:** Characterization of the CPE-loaded spray dried powders (yield %, DL% and LE% ± SE) and the resulting sublingual mini-tablets (weight and drug content ± SE; *n* = 6).

Sample	Spray-Dried Powders			Mini-Tablets	
	Yield %	DL%	LE%	Weight (mg)	RSV (mg)
**RSV-B/M-3**	73.2	13.40 ± 0.34	89.33 ± 2.27	30.43 ± 0.60	4.08 ± 0.08
**RSV-B/LYS-3**	74.8	15.15 ± 1.41	101.02 ± 9.39	31.18 ± 1.07	4.72 ± 0.16
**RSV-B/U-3**	75.4	15.03 ± 0.76	100.22 ± 5.07	30.20 ± 0.38	4.58 ± 0.06
**RSV-B/U-1**	83.2	11.02 ± 1.33	73.47 ± 8.87	29.11 ± 0.31	4.41 ± 0.05
**RSV-C/LYS-3**	73.2	15.75 ± 1.70	105.00 ± 11.33	31.38 ± 0.60	4.75 ± 0.09

**Table 8:** Extrapolated RSV biopharmaceutical parameters (Js, Kp, tlag, De and Ac ± SE) and RSV concentration in the donor chamber after CPE-loaded mini-tablets ex vivo permeation tests.

Sample	Js (μg/cm^2^∙h^-1^)	Kp (cm/h)	t_lag_ (min)	De (μg/cm^2^)	Ac (cm)	(RSV)_DONOR_ (μg/mL)
**RSV-B/M-3**	3.84 ± 1.61	0.08023 ± 0.05225	NO	14.28 ± 3.21	0.29870 ± 0.08721	47.82 ± 6.56
**RSV-B/LYS-3**	7.96 ± 1.00	0.16635 ± 0.03022	NO	2.84 ± 1.00	0.05941 ± 0.02586	47.89 ± 8.25
**RSV-B/U-3**	7.17 ± 1.77	0.05240 ± 0.01536	NO	23.94 ± 6.78	0.17504 ± 0.10061	136.76 ± 41.63
**RSV-B/U-1**	3.82 ± 0.18	0.05127 ± 0.00547	NO	14.36 ± 4.91	0.19267 ± 0.07104	74.55 ± 8.35
**RSV-C/LYS-3**	7.40 ± 0.96	0.22531 ± 0.02123	NO	5.90 ± 2.16	0.17979 ± 0.06396	32.83 ± 4.44

## Supplementary Materials

The following are available online at https://www.mdpi.com/article/10.3390/pharmaceutics13091370/s1, Figure S1: RSV permeation profile: amount of RSV permeated per unit area as a function of incubation time, Figure S2: RSV permeation profile in presence of sodium dodecyl sulfate as CPE (RSV:SDS = 5:1): amount of RSV permeated per unit area as a function of incubation time, Figure S3: RSV permeation profile in presence of sodium dehydrocolate as CPE (RSV:SDC = 5:1): amount of RSV permeated per unit area as a function of incubation time, Figure S4: RSV permeation profile in presence of Transcutol^®^ as CPE (RSV:T = 5:1): amount of RSV permeated per unit area as a function of incubation time, Figure S5: RSV permeation profile in presence of urea as CPE (RSV:U = 5:1): amount of RSV permeated per unit area as a function of incubation time, Figure S6: RSV permeation profile in presence of lysine as CPE (RSV:LYS = 5:1): amount of RSV permeated per unit area as a function of incubation time, Figure S7: RSV permeation profile in presence of menthol as CPE (RSV:M = 5:1): amount of RSV permeated per unit area as a function of incubation time, Figure S8: Appearance of A) RSV-A- and B) RSV-C-spray-dried powders, Figure S9: Appearance of RSV-loaded sublingual mini-tablets, Video S1: Visual disintegration assay of RSV-B mini-tablets (normal speed), Video S2: Visual disintegration assay of RSV-B mini-tablets (slow motion).
