# Supplementary material for: Improvement of Resveratrol Permeation through Sublingual Mucosa: Chemical Permeation Enhancers versus Spray Drying Technique to Obtain Fast-Disintegrating Sublingual Mini-Tablets
Source: Pharmaceutics. 2021 Aug 31;13(9):1370. doi: 10.3390/pharmaceutics13091370 (PMC8470294; doi:10.3390/pharmaceutics13091370)
Supplement: Supplementary file 1 [file pharmaceutics-13-01370-s001.zip › pharmaceutics-1320198-supplementary.pdf]

pharmaceutics

Supplementary Material: Improvement of Resveratrol Permeation through Sublingual Mucosa: Chemical Permeation Enhancers versus Spray Drying Technique to Obtain Fast-Disintegrating Sublingual Mini-Tablets

Giulia Di Prima, Giuseppe Angellotti, Amalia Giulia Scarpaci, Denise Murgia, Fabio D'Agostino, Giuseppina Campisi and Viviana De Caro

Data reported as supplementary material:

- **Figure S1:** RSV permeation profile: amount of RSV permeated per unit area as a function of incubation time
- **Figure S2:** RSV permeation profile in presence of Sodium Dodecyl Sulfate as CPE (RSV:SDS=5:1): amount of RSV permeated per unit area as a function of incubation time
- **Figure S3:** RSV permeation profile in presence of Sodium Dehydrocolate as CPE (RSV:SDC=5:1): amount of RSV permeated per unit area as a function of incubation time
- **Figure S4:** RSV permeation profile in presence of Transcutol® as CPE (RSV:T=5:1): amount of RSV permeated per unit area as a function of incubation time
- **Figure S5:** RSV permeation profile in presence of Urea as CPE (RSV:U=5:1): amount of RSV permeated per unit area as a function of incubation time
- **Figure S6:** RSV permeation profile in presence of Lysine as CPE (RSV:LYS=5:1): amount of RSV permeated per unit area as a function of incubation time
- **Figure S7:** RSV permeation profile in presence of Menthol as CPE (RSV:M=5:1): amount of RSV permeated per unit area as a function of incubation time
- **Figure S8:** Appearance of A) RSV-A and B) RSV-C spray dried powders
- **Figure S9:** Appearance of RSV-loaded sublingual mini-tablets
- **Video S1:** Visual disintegration assay of RSV-B mini-tablets (normal speed)
- **Video S2:** Visual disintegration assay of RSV-B mini-tablets (slow motion)

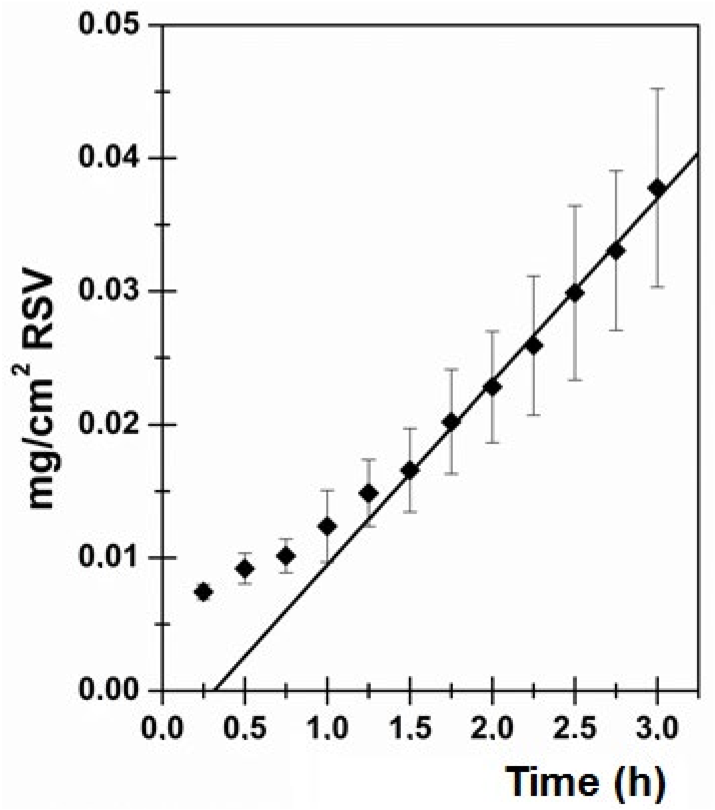

Figure S1. RSV permeation profile: amount of RSV permeated per unit area as a function of incubation time.

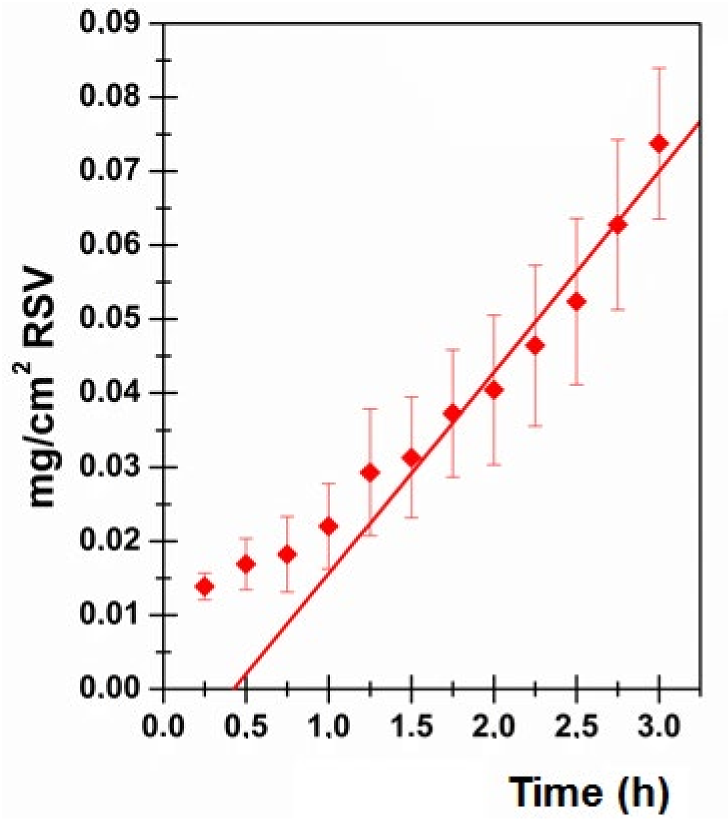

Figure S2. RSV permeation profile in presence of Sodium Dodecyl Sulfate as CPE (RSV:SDS=5:1): amount of RSV permeated per unit area as a function of incubation time.

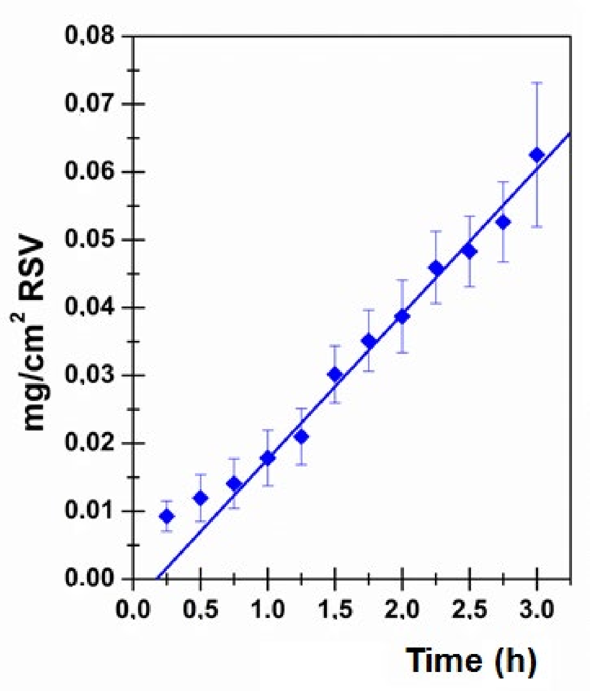

Figure S3. RSV permeation profile in presence of Sodium Dehydrocolate as CPE (RSV:SDC=5:1): amount of RSV permeated per unit area as a function of incubation time.

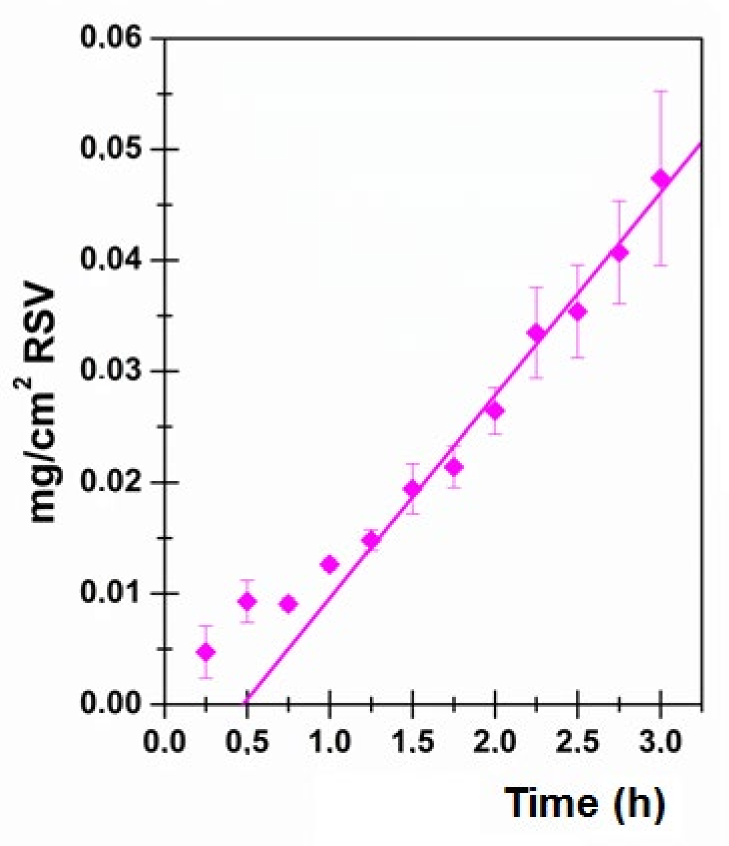

Figure S4. RSV permeation profile in presence of Transcutol® as CPE (RSV:T=5:1): amount of RSV permeated per unit area as a function of incubation time.

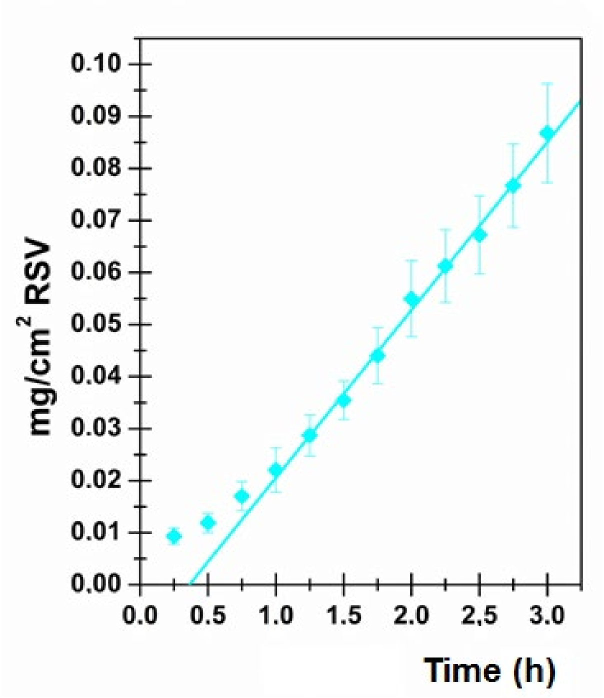

Figure S5. RSV permeation profile in presence of Urea as CPE (RSV:U=5:1): amount of RSV permeated per unit area as a function of incubation time.

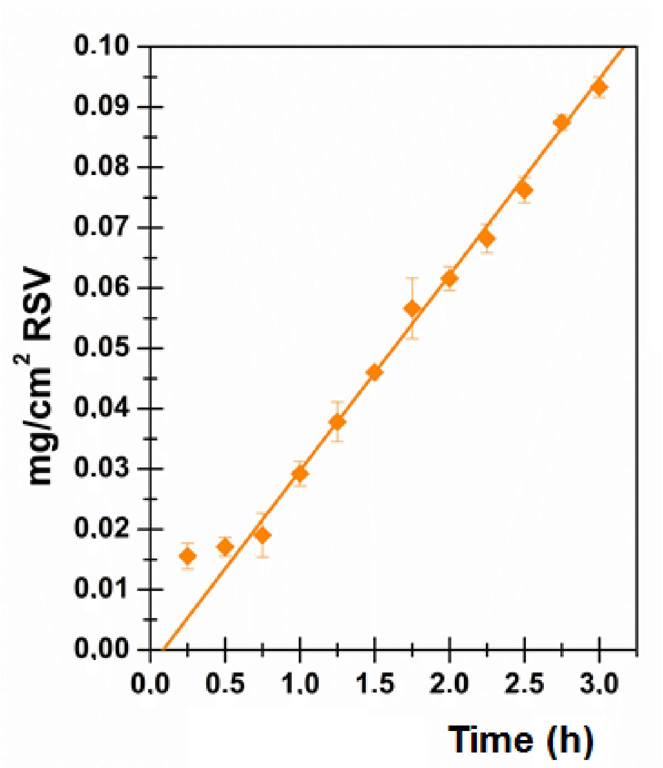

Figure S6. RSV permeation profile in presence of Lysine as CPE (RSV:LYS=5:1): amount of RSV permeated per unit area as a function of incubation time.

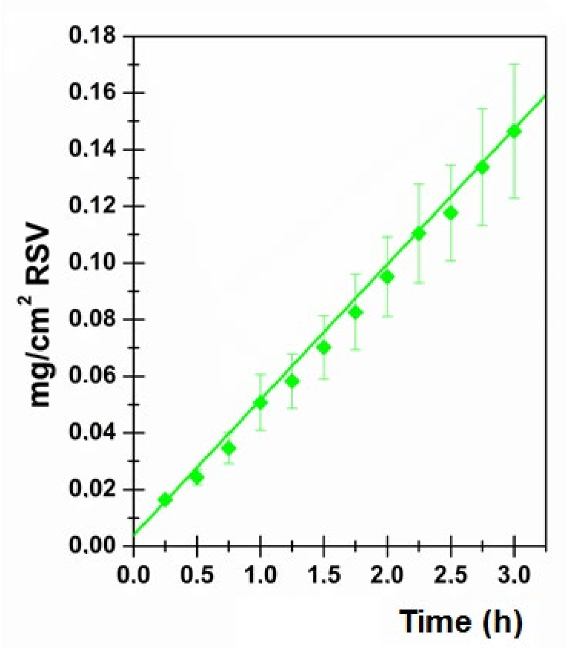

Figure S7. RSV permeation profile in presence of Menthol as CPE (RSV:M=5:1): amount of RSV permeated per unit area as a function of incubation time.

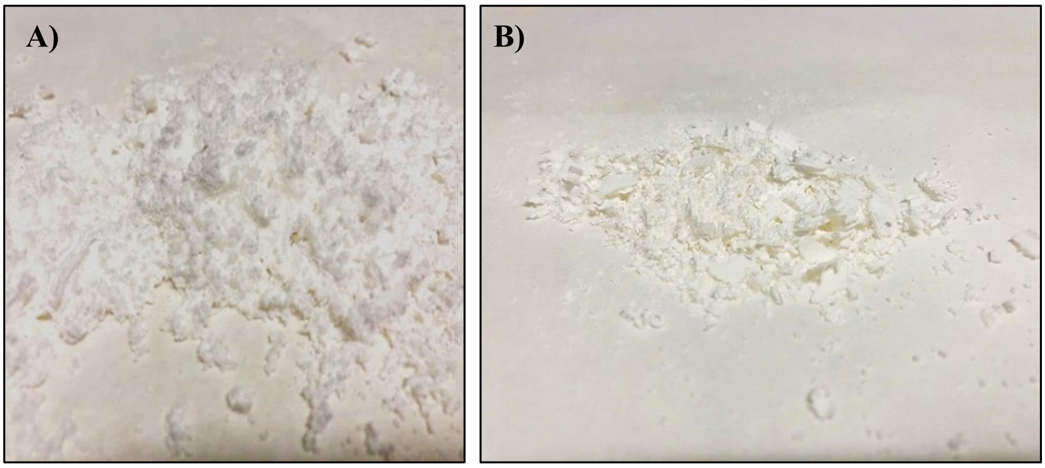

Figure S8. Appearance of A) RSV-A and B) RSV-C spray dried powders.

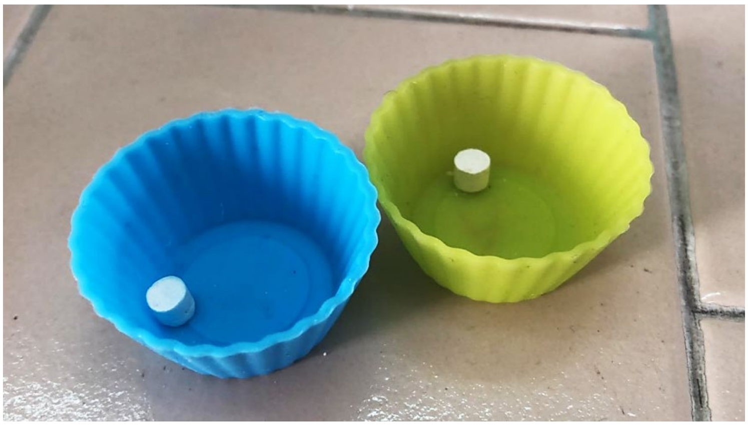

Figure S9. Appearance of RSV-loaded sublingual mini-tablets.
